# Multimodal MRI Deep Learning for Predicting Central Lymph Node Metastasis in Papillary Thyroid Cancer

**DOI:** 10.3390/cancers16234042

**Published:** 2024-12-02

**Authors:** Xiuyu Wang, Heng Zhang, Hang Fan, Xifeng Yang, Jiansong Fan, Puyeh Wu, Yicheng Ni, Shudong Hu

**Affiliations:** 1 Nurturing Center of Jiangsu Province for State Laboratory of AI Imaging & Interventional Radiology, Department of Radiology, Zhongda Hospital, Medical School, Southeast University, Nanjing 210018, China; 230249963@seu.edu.cn; 2 Department of Radiology, Affiliated hospital of Jiangnan University, Wuxi 214121, China; 9862022051@jiangnan.edu.cn; 3 School of Artificial Intelligence and Computer Science, Jiangnan University, Wuxi 214121, China; 6223110017@stu.jiangnan.edu.cn (H.F.); 6223112038@stu.jiangnan.edu.cn (X.Y.); 7233115007@stu.jiangnan.edu.cn (J.F.); 4 GE Healthcare, Beijing 100000, China; puyeh.wu@gehealthcare.com

**Keywords:** thyroid cancer, papillary thyroid cancer, central lymph node metastasis, magnetic resonance imaging, machine learning, deep learning

## Abstract

Background: Central lymph node metastasis (CLNM) in papillary thyroid cancer (PTC) significantly influences surgical decision-making strategies. Objectives: This study aims to develop a predictive model for CLNM in PTC patients using magnetic resonance imaging (MRI) and clinicopathological data. Methods: By incorporating deep learning (DL) algorithms, the model seeks to address the challenges in diagnosing CLNM and reduce overtreatment. The results were compared with traditional machine learning (ML) models. In this retrospective study, preoperative MRI data from 105 PTC patients were divided into training and testing sets. A radiologist manually outlined the region of interest (ROI) on MRI images. Three classic ML algorithms (support vector machine [SVM], logistic regression [LR], and random forest [RF]) were employed across different data modalities. Additionally, an AMMCNet utilizing convolutional neural networks (CNNs) was proposed to develop DL models for CLNM. Predictive performance was evaluated using receiver operator characteristic (ROC) curve analysis, and clinical utility was assessed through decision curve analysis (DCA). Results: Lesion diameter was identified as an independent risk factor for CLNM. Among ML models, the RF-(T1WI + T2WI, T1WI + T2WI + Clinical) models achieved the highest area under the curve (AUC) at 0.863. The DL fusion model surpassed all ML fusion models with an AUC of 0.891. Conclusions: A fusion model based on the AMMCNet architecture using MRI images and clinicopathological data was developed, effectively predicting CLNM in PTC patients.

## Simple Summary

Papillary thyroid cancer (PTC) is the most common subtype of thyroid cancer (TC), accounting for approximately 80–90% of all TC cases. PTC is generally considered a low-grade malignancy. However, aggressive behaviors, such as cervical lymph node metastasis, can be observed in some cases, with central lymph node metastasis (CLNM) regarded as the primary site of lymph node metastasis. In current clinical practice in China, prophylactic central lymph node dissection is commonly performed regardless of whether CLNM is present. This approach is associated with an increased risk of complications and unnecessary lymph node removal, raising concerns about overtreatment. Therefore, there is an urgent need for an efficient method to predict CLNM in PTC patients, which could guide clinical diagnosis and treatment strategies. In this study, we developed a fusion model based on the attention mechanism-based multimodal classification network (AMMCNet) architecture, integrating MRI images and clinicopathological data to efficiently predict CLNM in PTC patients.

## 1. Introduction

The global incidence of thyroid cancer (TC) has risen significantly over the past few decades [1]. Papillary thyroid carcinoma (PTC), accounting for 80–90% of all TC cases, is the most common subtype [2,3]. Despite generally a favorable prognosis with PTC, a number of patients experience aggressive forms of the disease, including cervical lymph node metastasis (LNM) [4]. Cervical LNM is strongly associated with higher rates of local recurrence and distant metastasis, negatively impacting disease-free survival (DFS) and overall survival (OS) rates [5,6].

Central lymph node metastasis (CLNM) is recognized as the primary site of cervical LNM in PTC [5,7]. However, current diagnostic methods for accurately detecting CLNM in PTC are inadequate. Ultrasonography (US), the predominant tool for preoperative lymph node assessment, exhibits variable sensitivity (26% to 47%) in identifying CLNM, making it insufficient for precise evaluation [8,9]. The effectiveness of US also heavily depends on the skill of the operator and is limited in imaging deeper anatomical structures [6]. Contrast-enhanced computed tomography (CT) provides enhancement over US by overcoming some of these limitations [6,10]. Nevertheless, both US and CT rely on morphological indicators to identify CLNM, which introduces subjectivity inherent in qualitative assessments. As a result, the detection rate for CLNM remains low, leading to misdiagnoses in 30% to 65% of PTC patients [11]. Due to this unsatisfactory detection rate, prophylactic central lymph node dissection (pCLND) is often recommended, despite the increased risks of complications such as recurrent laryngeal nerve injury and hypoparathyroidism, as well as potential overtreatment with unnecessary lymph node dissections [5,12]. Therefore, an accurate preoperative determination of CLNM status is critical.

Radiomics, an emerging technology, utilizes a high-throughput extraction of large-scale quantitative features from medical images and a machine learning (ML) algorithm for the classification [13]. Furthermore, based on convolutional neural networks (CNNs), the deep learning (DL) technique enables the automatic learning of crucial information from raw image data to perform tasks such as detection, classification, and segmentation [14]. A series of studies have been made regarding the application of ML radiomics and DL in thyroid disease research. Zhao et al. [15] developed ML radiomics models based on US and shear wave elastography images for thyroid nodules diagnosis and achieved a satisfactory diagnostic performance. Zhang et al. [16] used US-based ML radiomics, particularly the random forest classifier, demonstrating better thyroid nodules classification results than radiologists. Wang et al. [17] and Kwon et al. [18] constructed reliable US-based radiomics models for the prediction of extrathyroidal extension (ETE) and BRAF gene mutations, respectively. Similarly, Wu et al. [19] utilized US-based DL methods for the classification of thyroid nodules, demonstrating their effectiveness in improving diagnostic accuracy. Wang et al. [20] utilized CT-based DL methods for predicting cervical LNM in PTC, showcasing their potential to enhance preoperative diagnostic precision. Moreover, both ML radiomics and DL approaches are increasingly used in predicting CLNM in PTC [5,6,21].

Magnetic resonance imaging (MRI) is a non-invasive modality known for its outstanding soft tissue contrast. It is crucial for tumor detection, differentiation, evaluating treatment responses, and forecasting prognosis. MRI provides essential qualitative and quantitative insights at the cellular level, thus enhancing diagnostic and therapeutic outcomes [22]. Previous research has employed traditional ML radiomics methods based on MRI images to construct predictive models for LNM in PTC patients, achieving good predictive performance [22,23]. However, there are currently no studies using DL methods to develop predictive models for CLNM in PTC patients based on MRI images. According to previous research findings [24], DL methods can significantly enhance predictive performance compared to classical ML radiomics [25], which is primarily due to the ability of DL algorithms to capture complex information.

Therefore, we aim to develop a DL predictive model for CLNM in PTC patients based on multimodal MRI data and compare its performance with those of classical ML radiomics models.

## 2. Materials and Methods

### 2.1. Study Population and Clinical Pathological Characteristics

**Figure 1:**
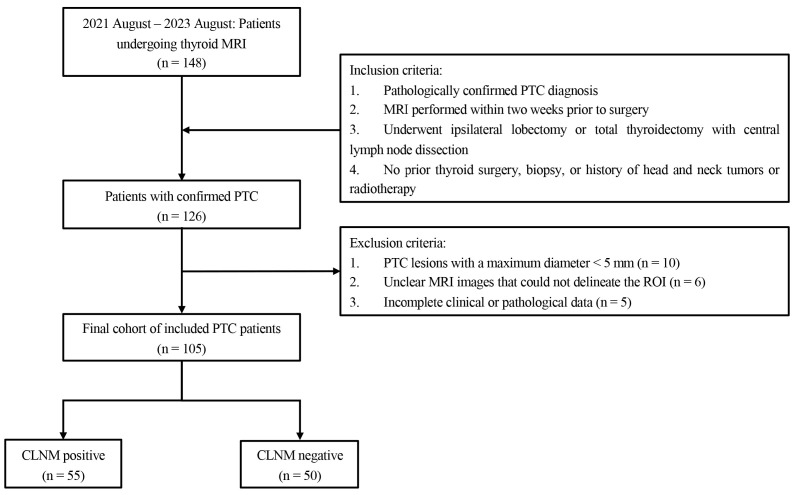
Inclusion and exclusion flowchart.

This retrospective study, approved by the Ethics Committee of Jiangnan University Affiliated Hospital (Approval Number: LS2020066), adheres to the principles of the Declaration of Helsinki. As this was a retrospective study, the requirement for patient informed consent was waived. Between August 2021 and August 2023, 148 patients who underwent thyroid MRI were initially assessed. Inclusion criteria included (1) PTC confirmed by surgical pathology; (2) MRI performed within two weeks prior to surgery; (3) patients who underwent ipsilateral lobectomy or total thyroidectomy with central lymph node dissection; (4) no prior thyroid surgery, biopsy, or history of head, neck tumors, or neck radiotherapy. Exclusion criteria included (1) PTC lesion with a maximum diameter less than 5 mm; (2) unclear MRI images that could not definitively delineate the region of interest (ROI); and (3) incomplete clinical or pathological data. The detailed patient selection process, including inclusion and exclusion criteria, is illustrated in Figure 1. Ultimately, 105 patients were included in the study with 55 individuals (52.38%) diagnosed as CLNM positive and 50 (47.62%) as CLNM negative. Patients were randomly assigned to training and testing sets in an 8:2 ratio for the construction of predictive models.

Clinical data including age and gender were collected, and pathology reports provided information on CLNM status, primary lesion diameter, extrathyroidal extension (ETE), multifocality, bilaterality, presence of calcification, and benign thyroid conditions.

### 2.2. MRI Protocol

MRI examinations were performed on a 3.0 Tesla MRI scanner (SIGNA Architect; GE Healthcare, Milwaukee, WI, USA) equipped with a 28-channel head and neck phased-array coil 1 to 2 weeks prior to surgery. During scanning, patients were positioned supine with their neck extended and shoulders relaxed downward to minimize artifacts in the clavicular area. Patients were also instructed to avoid swallowing during the scan. The scan coverage extended from the pharynx to the upper margin of the clavicle. The scanning protocols included axial T1-weighted imaging (T1WI), axial T2-weighted imaging (T2WI), axial diffusion-weighted imaging (DWI, with b-values of 0 and 500 s/mm^2^), and contrast-enhanced T1WI (CE-T1WI). T1WI was acquired using a spin echo sequence (Repetition Time/Echo Time [TR/TE] = 520/14 ms), and T2WI was acquired using a fast spin echo sequence (TR/TE = 3500/95 ms) with fat suppression. DWI was obtained using a single-shot echo-planar imaging sequence with short tau inversion recovery for fat suppression. Careful shimming was applied to correct local magnetic field inhomogeneities, optimizing image quality in the thyroid region. CE-T1WI Images with and without fat suppression were immediately acquired after the intravenous injection of 0.1 mmol/kg gadolinium-DTPA (Gd-DTPA) contrast agent at a flow rate of 1.5 mL/s (Magnevist; Schering AG, Berlin, Germany). Imaging parameters included a slice thickness of 3 mm, an interslice gap of 1 mm, a field of view (FOV) of 40 × 28 cm^2^, a matrix size of 256 × 256, and a number of excitation (NEX) of 4. The entire examination process was completed within 30 min.

### 2.3. ROI Segmentation

**Figure 2:**
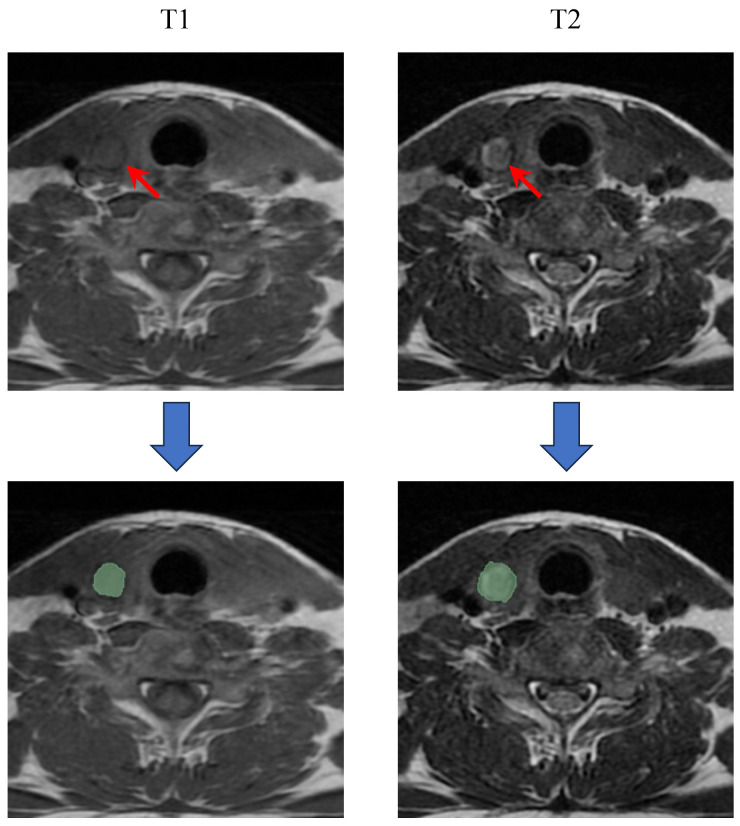
Segmentation of the ROI in axial T1 and T2 images. The red arrows in the MRI images indicate the location of the primary lesion.

MRI images from all patients were imported into 3D-Slicer software (version 5.2.2; http://www.slicer.org) for precise ROI segmentation (Figure 2). Following the previous research, for PTC patients with multiple lesions, the largest one was selected for analysis [9]. Initially, two radiologists determined the largest cross-sectional area of the primary tumor on T1WI and T2WI images in consensus. The ROI on the largest cross-sectional images was meticulously outlined by a junior radiologist (Observer 1, with 5 years of experience), carefully avoiding surrounding normal thyroid tissue. To ensure repeatability and consistency in the ROI delineation, intra-observer and inter-observer consistency checks were performed. Two weeks after the initial delineation, 30 cases were randomly selected for re-segmentation by Observer 1, and another experienced radiologist (Observer 2, with 10 years of experience) segmented the same 30 cases. Intra-observer and inter-observer consistency between segmentations was assessed by calculating the class correlation coefficient (CCC). A CCC greater than 0.75 indicates a high level of consistency in the ROI segmentation process. Figure 2 shows representative ROI delineations on T1WI and T2WI images.

### 2.4. Radiomics Feature Extraction and Selection

Data preprocessing included deduplication, normalization, and standardization. Subsequently, using the PyRadiomics package (version 3.0.1) in Python [26], radiomic features were extracted from T1WI and T2WI images and categorized into three main groups: first-order features, shape features, and texture features. Texture features include (I) Gray Level Size Zone Matrix (GLSZM); (II) Gray Level Run-Length Matrix (GLRLM); (III) Gray Level Dependence Matrix (GLDM); (IV) Neighborhood Gray-Tone Difference Matrix (NGTDM); (V) Gray Level Co-occurrence Matrix (GLCM). Additionally, higher-order radiomic features were extracted from images processed with wavelet or logarithmic transformations.

**Figure 3:**
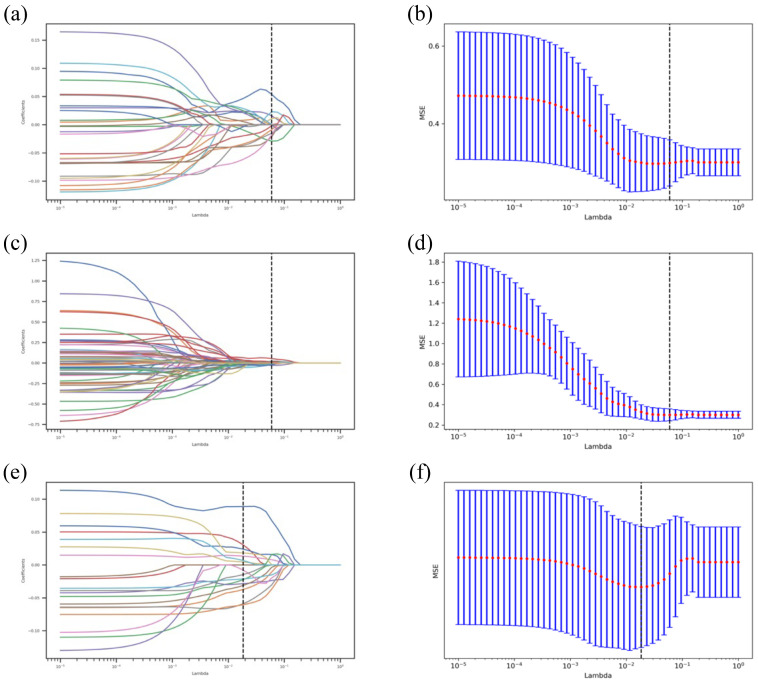
Distribution of LASSO coefficients for T1 features, T2 features, and combined T1 + T2 features. (**a**,**b**) represent T1 features, (**c**,**d**) represent T2 features and (**e**,**f**) represent the combined T1 + T2 features.

For T1WI features, we conducted a detailed selection process. Firstly, we used the Mann–Whitney U test to retain features with a *p*-value less than 0.05, which was followed by further selection using the Pearson correlation coefficient (PCC). For features that were selected, we employed the Least Absolute Shrinkage and Selection Operator (LASSO) method for regression analysis (Figure 3). The LASSO method incrementally shrinks regression coefficients by adjusting the penalty parameter λ until they reach zero, effectively setting the coefficients of most irrelevant features to zero. Using 10-fold cross-validation, we identified the optimal λ value to minimize the cross-validation error and ultimately obtained the best features. The same approach was applied for T2WI features. For combined T1 + T2 features, we performed a multi-layer selection. Initially, we retained features with a *p*-value less than 0.05 using the Mann–Whitney U test, which was followed by further selection using PCC. Features were then further selected using the Maximum Relevance Minimum Redundancy (mRMR) algorithm and the LASSO algorithm (Figure 3). The mRMR algorithm aims to maximize the relevance between the features and the classification variable while minimizing the redundancy among the features, thereby eliminating all redundant and irrelevant features to ultimately obtain the best features.

### 2.5. Construction and Validation of ML Radiomics Models

ML predictive models were constructed by integrating radiomic features and clinical information. Initially, models based solely on radiomic features were developed using support vector machine (SVM), logistic regression (LR), and random forest (RF) for T1WI models, T2WI models, and combined T1WI + T2WI models. Following this, we combined imaging features with clinical information to construct SVM, LR, and RF models for T1WI + Clinical, T2WI + Clinical, and comprehensive T1WI + T2WI + Clinical models. The predictive performance and clinical utility of these models were assessed on the test data set using receiver operating characteristic (ROC) curve analysis and decision curve analysis (DCA).

### 2.6. Construction and Validation of DL Models

**Figure 4:**
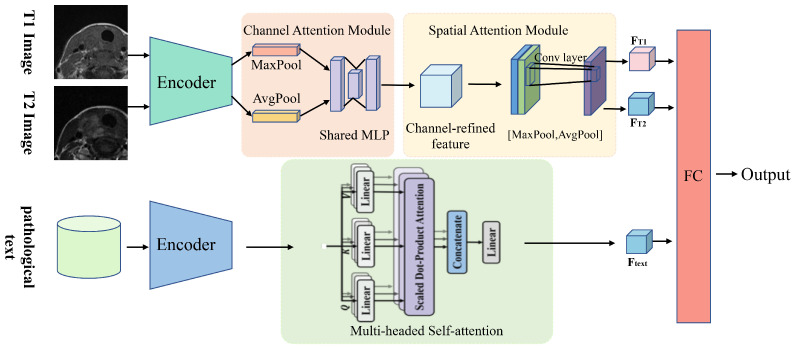
Deep learning model workflow.

We proposed an attention mechanism-based multimodal classification network (AMMCNet), a sophisticated DL architecture integrating CNN and attention mechanisms, specifically designed for processing and analyzing MRI images and clinical pathology text information (Figure 4). This architecture consists of three main components: image feature extraction, text feature extraction, and feature fusion module. The network utilizes channel and spatial attention mechanisms for image feature extraction, extracting hierarchical features through a series of convolution and pooling layers. Text information is processed through a sequential module, including linear layers and ReLU activation, with a transformer encoder capturing contextual information and long-range dependencies within the text data. The feature fusion stage combines features from both image and text modalities processed through a linear layer to reduce their dimensionality, allowing the effective integration of complementary information for better classification. Predictive models were developed utilizing the AMMCNet classification network, which was initially based on imaging features (DL-T1, DL-T2, and DL-T1 + T2 models). Subsequently, we combined imaging features with clinical information to construct DL-T1 + Clinical, DL-T2 + Clinical, and the comprehensive fusion models. The predictive performance of DL models was evaluated on the test set using ROC and DCA analyses.

### 2.7. Statistical Analysis

Continuous variables from clinical pathology data were analyzed using the independent samples *t*-test or the Mann–Whitney U test, while categorical variables were assessed through the chi-square test or Fisher’s exact test. All statistical analyses were conducted using R software (version 4.3.0; http://www.Rproject.org). The performance of models was evaluated using ROC curve analysis, yielding the area under the curve (AUC), and the accuracy (ACC), sensitivity (SEN), specificity (SPE), positive predictive value (PPV), and negative predictive value (NPV) were recorded.

## 3. Results

### 3.1. Clinical Baseline Characteristics

A total of 105 patients (28 males and 77 females, age: 46.19 ± 11.66 years, age range: 24–74 years) were included in the study. Data from these patients were divided into a training set (n = 84) and a test set (n = 21) according to an 8:2 ratio. The clinical baseline characteristics of the CLNM positive and CLNM negative groups are shown in Table 1. In the univariate analysis, both lesion diameter (OR = 1.966, *p* = 0.019) and ETE status (OR = 2.227, *p* = 0.045) showed significant differences. Further multivariate logistic regression analysis identified lesion diameter as a significant independent risk factor for predicting CLNM in PTC patients (*p* < 0.05) (Table 2). Table 3 displays the distribution of clinical baseline characteristics in the training and test sets, with no significant differences noted.

### 3.2. Radiomics Feature Extraction and Selection

**Figure 5:**
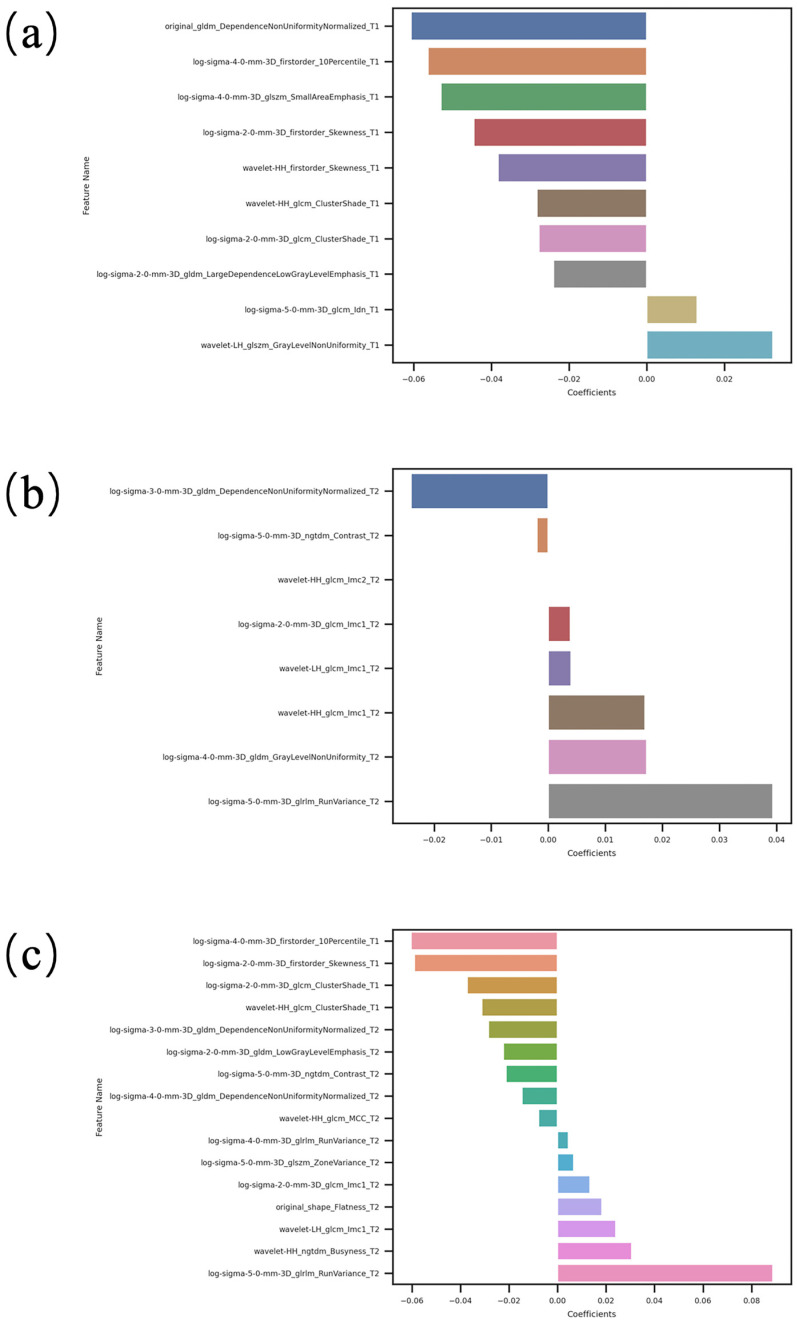
Histograms of best feature coefficients for T1, T2, and T1 + T2. (**a**) The best feature coefficients for T1; (**b**) the best feature coefficients for T2; (**c**) the best feature coefficients for T1 + T2.

From both T1WI and T2WI images, 864 radiomic features were extracted. For T1WI radiomic features, the Mann–Whitney U test reduced this to 41 features, and PCC further narrowed it down to 28 features, with 10 best features selected after LASSO. Regarding T2WI radiomic features, 90 features remained after the Mann–Whitney U test, 59 features after PCC, and 8 best features after LASSO. For combined T1WI + T2WI radiomic features, 94 features remained after the Mann–Whitney U test, 63 features after PCC, 20 features after mRMR, and finally, the 16 best features were selected after LASSO (Figure 5 and Table 4).

### 3.3. Performance of ML Radiomics Models

**Figure 6:**
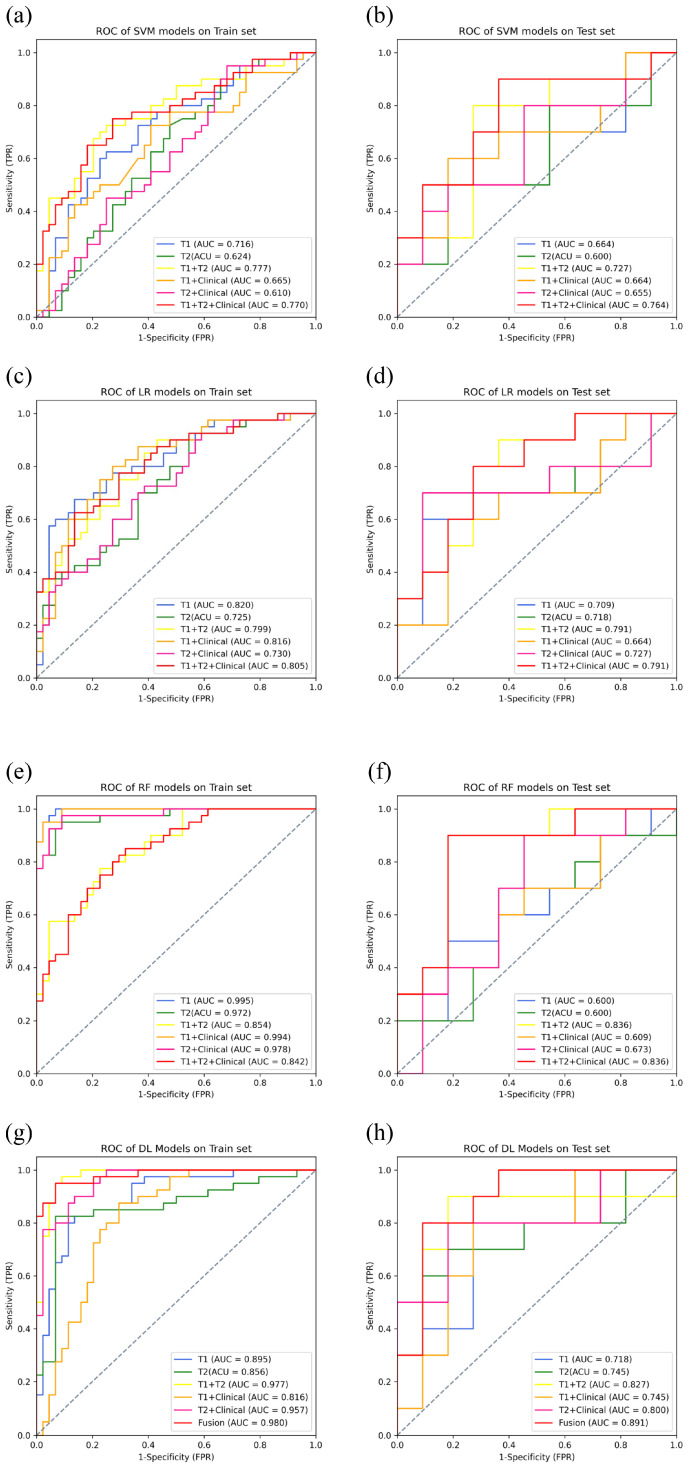
The ROC curves of ML and DL models on the training set and test set. (**a**,**b**) The ROC curves of the SVM models on the training set and test set, (**c**,**d**) the ROC curves of the LR models on the training set and test set, (**e**,**f**) the ROC curves of RF models on training set and test set, (**g**,**h**) ROC curves of DL models on training set and test set.

**Figure 7:**
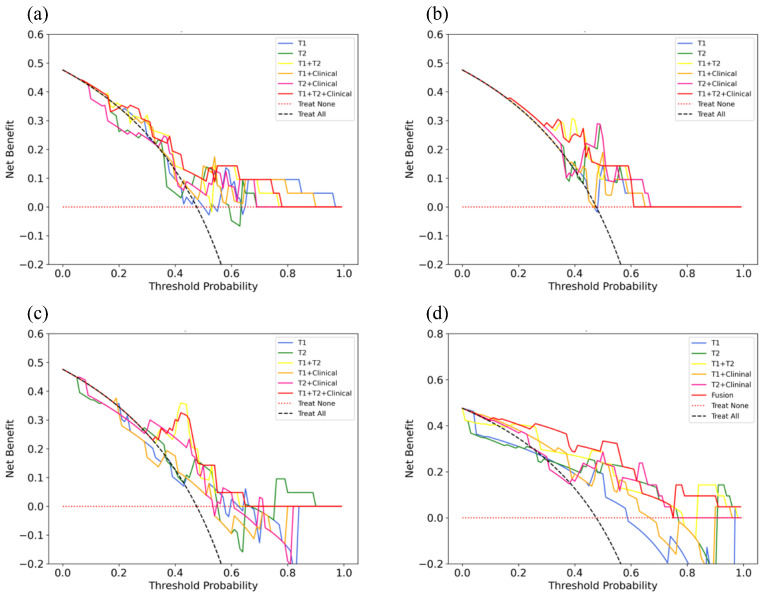
ML and DL models’ DCA curves on the test set. (**a**) The DCA curves of the SVM models on the test set, (**b**) the DCA curves of the LR models on the test set, (**c**) the DCA curves of RF models on the test set, and (**d**) the DCA curves of the DL modes on the test set.

Table 5 and Figure 6 display the performance of SVM models in predicting CLNM in PTC. For AUC, the highest AUC was achieved by the T1WI + T2WI + Clinical model (0.764), and the lowest by the T2 model (0.600). For ACC, the T1WI, T1WI + T2WI, and T1WI + T2WI + Clinical models had the highest accuracy (0.714), while the T2 model had the lowest (0.476). For SEN, the T1WI, T1WI + T2WI, T1WI + Clinical, and T1WI + T2WI + Clinical models achieved the highest sensitivity (0.700), and the T2 model had the lowest (0.500). For SPE, the highest specificity was observed in the T1WI, T1WI + T2WI, and T1WI + T2WI + Clinical models (0.727), and the T2 model had the lowest (0.455). For PPV, the T1WI, T1WI + T2WI, and T1 + T2 + Clinical models had the highest values (0.700), and the T2 model the lowest (0.455). Finally, for NPV, the highest was found in the T1, T1 + T2, and T1 + T2 + Clinical models (0.727), and the lowest in the T2 model (0.500). The results of the DCA show that only the T1 + T2 + Clinical model briefly falls below the extreme curve (Treat all), while other models are consistently below both extreme curves (Treat all and Treat none) to varying degrees. This indicates that the T1 + T2 + Clinical model has a higher clinical utility (Figure 7a). Table 6 and Figure 6 present the performance of the LR models in predicting CLNM in PTC. For AUC, within the test set, the T1 + T2 and T1 + T2 + Clinical models showed the highest AUC (0.791), while the T1 + Clinical model had the lowest (0.664). In terms of ACC, the highest accuracy was recorded by the T1 + T2 and T1 + T2 + Clinical models (0.762), and the lowest by the T1 + Clinical model (0.667). For SEN, the T1 + T2 and T1 + T2 + Clinical models achieved the highest sensitivity (0.800), and the lowest sensitivity was seen in the T1, T2, T1 + Clinical, and T2 + Clinical models (0.700). Regarding SPE, the highest specificity was observed in the T1, T2, T1 + T2, T2 + Clinical, and T1 + T2 + Clinical models (0.727), with the T1 + Clinical model recording the lowest (0.636). For PPV, the highest values were found in the T1 + T2 and T1 + T2 + Clinical models (0.727), and the lowest in the T1 + Clinical model (0.636). Finally, for NPV, the highest was in the T1 + T2 and T1 + T2 + Clinical models (0.800), and the lowest in the T1 + Clinical model (0.700). The results of the DCA indicate that only the T1 + T2 and T1 + T2 + Clinical models did not fall below the extreme curve. In contrast, other models did fall below the extreme curve. Moreover, the T1 + T2 + Clinical model performed better than the T1 + T2 model across most threshold levels, suggesting that the T1 + T2 + Clinical model has superior clinical utility (Figure 7b). Table 7 and Figure 6 shows the performance of the RF models in predicting CLNM in PTC. For AUC, within the test set, the highest AUC was recorded by the T1 + T2 and T1 + T2 + Clinical models (0.836), while the lowest AUC was observed in the T2 model (0.600). In terms of ACC, the highest accuracy was achieved by the T1 + T2 model (0.857), and the lowest accuracy was seen in the T1 and T2 + Clinical models (0.571). For SEN, the highest sensitivity was shown by the T1 + T2 model (0.900), and the lowest sensitivity by the T2 + Clinical model (0.500). Regarding SPE, the T1 + T2 and T1 + T2 + Clinical models had the highest specificity (0.818), while the T1 model recorded the lowest (0.545). For PPV, the highest PPV was found in the T1 + T2 model (0.818), and the lowest in the T1 model (0.546). Finally, for NPV, the highest NPV was in the T1 + T2 model (0.900), and the lowest in the T2 + Clinical model (0.583). DCA analysis revealed that only the T1WI + T2WI and T1WI + T2WI + Clinical models did not fall below the extreme curves. Additionally, the T1WI + T2WI + Clinical model consistently outperformed the T1WI + T2WI model across most threshold levels, indicating its higher clinical utility (Figure 7c).

### 3.4. Performance of DL Models

Table 8 and Figure 6 display the performance of DL models in predicting CLNM in PTC. For AUC, the highest AUC was achieved by the fusion model (0.891), while the lowest was recorded by the T1WI model (0.718). For ACC, the highest accuracy was observed in the T1WI + T2WI and fusion models (0.857), with the lowest accuracy found in the T1WI model (0.714). For SEN, the highest sensitivity was shown in the T1WI + T2WI model (0.900), and the lowest was in the T1WI and T2WI models (0.700). For SPE, the highest specificity was recorded by the fusion model (0.909), and the lowest was in the T1WI and T1WI + Clinical models (0.727). For PPV, the highest PPV was achieved by the fusion model (0.889), and the lowest was in the T1 model (0.700). Finally, for NPV, the highest NPV was in the T1 + T2 model (0.900), and the lowest was in the T1 model (0.727). DCA results demonstrate that only the fusion model did not fall below the extreme curve, suggesting that the integrated model holds greater clinical utility (Figure 7d).

## 4. Discussion

In this study, we developed a fusion model integrating T1WI and T2WI images with clinical pathological data using the proposed DL architecture, AMMCNet, to effectively predict CLNM in patients with PTC. This model demonstrated superior predictive performance compared to traditional ML models, indicating the potential advantages of DL-based models in this context. Beyond its technical contributions, the proposed model has significant clinical implications. By providing a more accurate preoperative prediction of CLNM, it has the potential to reduce overtreatment in PTC patients. Specifically, it can guide more precise surgical decision making, such as avoiding unnecessary pCLND, which are associated with higher risks of complications. This highlights the model’s potential to improve patient outcomes and optimize resource utilization in clinical practice.

Univariate analysis revealed significant differences in lesion diameter and ETE between the CLNM-positive and CLNM-negative groups (*p* < 0.05). Multivariate logistic regression analysis further highlighted lesion diameter (OR = 1.137, *p* < 0.01) as an independent clinical risk factor related to CLNM in PTC patients. Previous studies have identified lesion diameter, ETE, age, gender, and multifocality as independent risk factors related to CLNM [9]. Our findings partly align with their results, which was potentially due to a small sample size. Future studies should consider expanding the sample size to uncover more significant clinical features, allowing for the further refinement and optimization of current models.

The radiomics features in this study included first-order statistics, morphological features, and texture features, including GLSZM, GLRLM, GLDM, NGTDM, and GLCM. Analysis of the T1WI, T2WI, and T1WI + T2WI features revealed that the majority of the best features were texture features, which assess tumor heterogeneity by analyzing the grayscale distribution in images at different scales and directions. Tumor heterogeneity, a fundamental characteristic of malignancies, significantly affects tumor growth, invasiveness, drug response, and prognosis [27]. Assessment of tissue heterogeneity has been a major focus in oncological research, with genomic studies confirming its close association with biological prognosis, posing challenges for treatment strategies. Radiomics features have shown a strong correlation with tumor heterogeneity at the molecular level [28].

The results of this study indicated that the RF-T1WI + T2WI model’s AUC was slightly higher than the DL-T1WI + T2WI model (0.863 vs. 0.827). However, DL models generally exhibited higher AUCs compared to the corresponding ML models. Recent reviews comparing the performance of DL models, classic ML models, and multi-domain fusion models in medical research have shown that in 65% of the studies, DL models outperformed ML models, while in 20% they performed worse, and in 15% they showed comparable performance [29]. Our comparative analysis confirmed that DL models are superior in predicting CLNM compared to traditional ML methods, which is consistent with the most prior results. Studies on predicting occult lymph node metastasis in laryngeal squamous cell carcinoma and classifying colorectal cancer lymph node metastasis also support that DL models offer enhanced predictive performance, facilitating a better integration of AI algorithms with medical diagnostics [9,30,31].

For ML models, the SVM-T1WI + T2WI + Clinical model achieved the highest AUC (0.764) among the SVM models, the LR-(T1WI + T2WI, T1WI + T2WI + Clinical) models achieved the highest AUC (0.791) among the LR models, and the RF-(T1WI + T2WI, T1WI + T2WI + Clinical) models achieved the highest AUC (0.836) among the RF models. In the SVM models, the AUC for T1WI + T2WI + Clinical was slightly higher than that for T1WI + T2WI, whereas in the LR and RF models, the AUCs for T1WI + T2WI and T1WI + T2WI + Clinical were similar, which was likely because lesion diameter was the only clinical feature included. Thus, the improvement from the T1WI + T2WI + Clinical model over the T1WI + T2WI model was minimal. Moreover, our study found that the AUC of the RF-T1WI + T2WI + Clinical model was significantly higher than that of the SVM and LR models using the same data, which was consistent with previous findings [32]. This may be due to RF’s ability to build numerous decision trees from random subsets of training data and features, thereby enhancing model robustness. The RF model, considered an ensemble of decision trees, benefits from MRI image data by reducing information loss as the tree depth increases [31]. Overall, MRI images combined with clinical pathology data can serve as effective predictors of CLNM, but selecting the appropriate algorithm is crucial.

The integration of the proposed model into clinical practice requires addressing several practical challenges. Key barriers include regulatory compliance, training requirements for radiologists to effectively interpret model outputs, and the cultural acceptance of AI-driven tools within the medical community. To overcome these challenges, multi-center validation studies and targeted training programs are essential to build trust and facilitate adoption. These steps will enhance the model’s clinical applicability and ensure its effective integration into routine practice.

Despite the meticulous design and implementation of this study, there are some limitations. First, the small sample size, especially in the test set, may limit the generalizability of the predictive models. Secondly, all data in this study came from a single center, lacking external validation. Additionally, for multifocal PTC, we only included the largest lesion and extracted features from the primary tumor, ignoring information from lymph nodes, which may affect predictive performance. Finally, due to MRI resolution limitations, lesions smaller than 5 mm in diameter were excluded, potentially limiting the comprehensiveness of the conclusions.

## 5. Conclusions

We developed a fusion model based on the AMMCNet architecture, integrating MRI images and clinicopathological data to efficiently detect CLNM in PTC patients. The model significantly outperforms ML models and has the potential to assist clinicians to make more accurate treatment decisions and to prevent overtreatment in PTC patients.

## Figures and Tables

**Table 1:** Baseline of CLNM positive group and CLNM negative group.

Characteristics	CLNM (+) (n = 50)	CLNM (−) (n = 55)	*p* Value
Age, Mean ± SD	44.62 ± 11.68	47.62 ± 11.57	0.190
Diameter, M (Q_1_, Q_3_)	1.15 (0.80, 1.65)	1.00 (0.60, 1.50)	0.037 *
Gender, n (%)			0.105
Male	17 (34.00)	11 (20.00)	
Female	33 (66.00)	44 (80.00)	
ETE, n (%)			0.044 *
Yes	28 (56.00)	20 (36.36)	
No	22 (44.00)	35 (63.64)	
Multifocal, n (%)			0.163
Yes	17 (34.00)	12 (21.82)	
No	33 (66.00)	43 (78.18)	
Biliteral, n (%)			0.150
Yes	14 (28.00)	9 (16.36)	
No	36 (72.00)	46 (83.64)	
Calcification, n (%)			0.679
Yes	1 (2.00)	3 (5.45)	
No	49 (98.00)	52 (94.55)	
Benign lesions, n (%)			0.654
Yes	26 (52.00)	31 (56.36)	
No	24 (48.00)	24 (43.64)	

PTC: papillary thyroid cancer; CLNM: central lymph node metastasis; ETE: extrathyroidal extension; *: *p* < 0.05.

**Table 2:** Univariate analysis and multivariate logistic regression analysis.

	OR (95% CI)	*p* Value
Age	0.937 (0.864–1.017)	0.190
Diameter	1.137 (1.050–1.231)	0.008 *
Gender	1.083 (0.998–1.174)	0.107
ETE	1.104 (1.018–1.196)	0.044 *
Multifocality	1.071 (0.987–1.162)	0.166
Bilateral	1.073 (0.980–1.164)	0.153
Calcification	0.956 (0.881–1.038)	0.361
Benign lesion	0.978 (0.881–1.038)	0.658

ETE: extrathyroidal extension; CI: confidence interval; OR: odds ratio; *: *p* < 0.05.

**Table 3:** Distribution of baseline between training and test sets.

Characteristics	Training Cohort (n = 84)	Test Cohort (n = 21)	*p* Value
Age, Mean ± SD	46.38 ± 11.91	45.43 ± 10.84	0.740
Diameter, M (Q_1_, Q_3_)	1.10 (0.70–1.50)	0.80 (0.60–1.80)	0.782
CLNM, n (%)			0.329
Positive	42 (50.00)	8 (38.10)	
Negative	42 (50.00)	13 (61.90)	
Gender, n (%)			0.741
Male	23 (27.38)	5 (23.81)	
Female	61 (72.62)	16 (76.19)	
ETE, n (%)			0.493
Yes	37 (44.05)	11 (52.38)	
No	47 (55.95)	10 (47.62)	
Multifocal, n (%)			0.913
Yes	23 (27.38)	6 (28.57)	
No	61 (72.62)	15 (71.43)	
Biliteral, n (%)			1.000
Yes	18 (21.43)	5 (23.81)	
No	66 (78.57)	16 (76.19)	
Calcification, n (%)			0.581
Yes	4 (4.76)	0 (0.00)	
No	80 (95.24)	21 (100.00)	
Benign lesions, n (%)			0.433
Yes	44 (52.38)	13 (61.90)	
No	40 (47.62)	8 (38.10)	

CLNM: central lymph node metastasis; ETE: extrathyroidal extension.

**Table 4:** Best radiomic features of T1, T2 and T1 + T2.

Sequence	Feature Name
Best T1 features	original_gldm_DependenceNonUniformityNormalized
	log-sigma-2-0-mm-3D_firstorder_Skewness
	log-sigma-2-0-mm-3D_glcm_ClusterShade
	log-sigma-2-0-mm-3D_gldm_LargeDependenceLowGrayLevelEmphasis
	log-sigma-4-0-mm-3D_firstorder_10Percentile
	log-sigma-4-0-mm-3D_glszm_SmallAreaEmphasis
	log-sigma-5-0-mm-3D_glcm_Idn
	wavelet-LH_glszm_GrayLevelNonUniformity
	wavelet-HH_firstorder_Skewness
	wavelet-HH_glcm_ClusterShade
Best T2 features	log-sigma-2-0-mm-3D_glcm_Imc1
	log-sigma-3-0-mm-3D_gldm_DependenceNonUniformityNormalized
	log-sigma-4-0-mm-3D_gldm_GrayLevelNonUniformity
	log-sigma-5-0-mm-3D_glrlm_RunVariance
	log-sigma-5-0-mm-3D_ngtdm_Contrast
	wavelet-LH_glcm_Imc1
	wavelet-HH_glcm_Imc1
	wavelet-HH_glcm_Imc2
Best T1 + T2 features	log-sigma-2-0-mm-3D_glcm_ClusterShade
	wavelet-HH_glcm_ClusterShade
	log-sigma-2-0-mm-3D_firstorder_Skewness
	log-sigma-4-0-mm-3D_firstorder_10Percentile
	log-sigma-5-0-mm-3D_glrlm_RunVariance
	log-sigma-2-0-mm-3D_gldm_LowGrayLevelEmphasis
	wavelet-HH_ngtdm_Busyness
	log-sigma-3-0-mm-3D_gldm_DependenceNonUniformityNormalized
	log-sigma-2-0-mm-3D_glcm_Imc1
	log-sigma-4-0-mm-3D_glrlm_RunVariance
	log-sigma-5-0-mm-3D_ngtdm_Contrast
	wavelet-LH_glcm_Imc1
	wavelet-HH_glcm_MCC
	log-sigma-4-0-mm-3D_gldm_DependenceNonUniformityNormalized
	original_shape_Flatness
	log-sigma-5-0-mm-3D_glszm_ZoneVariance

**Table 5:** Performance of SVM models on training and test sets.

SVM Models	Set	AUC (95% CI)	ACC	SEN	SPE	PPV	NPV
T1	Training	0.716 (0.599–0.821)	0.655	0.425	0.864	0.739	0.623
T2	Training	0.624 (0.505–0.744)	0.571	0.825	0.341	0.532	0.682
T1 + T2	Training	0.777 (0.663–0.876)	0.679	0.800	0.568	0.628	0.758
T1 + Clinical	Training	0.665 (0.553–0.779)	0.631	0.425	0.818	0.680	0.610
T2 + Clinical	Training	0.610 (0.494–0.730)	0.607	0.875	0.364	0.556	0.762
T1 + T2 + Clinical	Training	0.770 (0.664–0.868)	0.690	0.775	0.614	0.646	0.750
T1	Test	0.664 (0.398–0.900)	0.714	0.700	0.727	0.700	0.727
T2	Test	0.600 (0.324–0.852)	0.476	0.500	0.455	0.455	0.500
T1 + T2	Test	0.727 (0.479–0.935)	0.714	0.700	0.727	0.700	0.727
T1 + Clinical	Test	0.664 (0.418–0.885)	0.667	0.700	0.636	0.636	0.700
T2 + Clinical	Test	0.655 (0.391–0.889)	0.571	0.600	0.545	0.546	0.600
T1 + T2 + Clinical	Test	0.764 (0.510–0.971)	0.714	0.700	0.727	0.700	0.727

CI: confidence interval; AUC: area under curve; ACC: accuracy; SEN: sensitivity; SPE: specificity; PPV: positive predictive value; NPV: negative predictive value.

**Table 6:** Performance of LR models on training and test sets.

LR Models	Set	AUC (95% CI)	ACC	SEN	SPE	PPV	NPV
T1	Training	0.820 (0.724–0.905)	0.762	0.675	0.841	0.794	0.740
T2	Training	0.725 (0.609–0.829)	0.667	0.700	0.636	0.636	0.700
T1 + T2	Training	0.799 (0.707–0.886)	0.655	0.900	0.432	0.590	0.826
T1 + Clinical	Training	0.816 (0.704–0.905)	0.738	0.675	0.795	0.750	0.729
T2 + Clinical	Training	0.730 (0.617–0.831)	0.631	0.725	0.545	0.592	0.686
T1 + T2 + Clinical	Training	0.805 (0.704–0.892)	0.690	0.900	0.500	0.621	0.846
T1	Test	0.709 (0.472–0.926)	0.714	0.700	0.727	0.700	0.727
T2	Test	0.718 (0.469–0.945)	0.714	0.700	0.727	0.700	0.727
T1 + T2	Test	0.791 (0.548–0.963)	0.762	0.800	0.727	0.727	0.800
T1 + Clinical	Test	0.664 (0.417–0.904)	0.667	0.700	0.636	0.636	0.700
T2 + Clinical	Test	0.727 (0.455–0.959)	0.714	0.700	0.727	0.700	0.727
T1 + T2 + Clinical	Test	0.791 (0.577–0.962)	0.762	0.800	0.727	0.727	0.800

CI: confidence interval; AUC: area under curve; ACC: accuracy; SEN: sensitivity; SPE: specificity; PPV: positive predictive value; NPV: negative predictive value.

**Table 7:** Performance of LR models on training and test sets.

RF Models	Set	AUC (95% CI)	ACC	SEN	SPE	PPV	NPV
T1	Training	0.995 (0.985–1.000)	0.952	0.950	0.955	0.950	0.955
T2	Training	0.972 (0.936–0.997)	0.917	0.950	0.886	0.884	0.951
T1 + T2	Training	0.854 (0.773–0.929)	0.726	0.875	0.591	0.660	0.839
T1 + Clinical	Training	0.994 (0.981–1.000)	0.952	0.925	0.977	0.974	0.935
T2 + Clinical	Training	0.978 (0.949–0.998)	0.940	0.925	0.955	0.949	0.933
T1 + T2 + Clinical	Training	0.842 (0.754–0.918)	0.714	0.850	0.591	0.654	0.813
T1	Test	0.600 (0.357–0.847)	0.571	0.600	0.545	0.546	0.600
T2	Test	0.600 (0.333–0.866)	0.667	0.700	0.636	0.636	0.700
T1 + T2	Test	0.836 (0.618–1.000)	0.857	0.900	0.818	0.818	0.900
T1 + Clinical	Test	0.609 (0.327–0.846)	0.619	0.600	0.636	0.600	0.636
T2 + Clinical	Test	0.673 (0.422–0.900)	0.571	0.500	0.636	0.556	0.583
T1 + T2 + Clinical	Test	0.836 (0.611–1.000)	0.810	0.800	0.818	0.800	0.818

CI: confidence interval; AUC: area under curve; ACC: accuracy; SEN: sensitivity; SPE: specificity; PPV: positive predictive value; NPV: negative predictive value.

**Table 8:** Performance of DL models on training and test sets.

DL Models	Set	AUC (95% CI)	ACC	SEN	SPE	PPV	NPV
T1	Training	0.895 (0.826–0.963)	0.845	0.825	0.864	0.846	0.844
T2	Training	0.856 (0.767–0.945)	0.881	0.825	0.932	0.917	0.854
T1 + T2	Training	0.977 (0.950–1.000)	0.940	0.975	0.909	0.907	0.976
T1 + Clinical	Training	0.816 (0.723–0.910)	0.786	0.875	0.705	0.729	0.861
T2 + Clinical	Training	0.957 (0.920–0.994)	0.881	0.900	0.864	0.857	0.905
Fusion	Training	0.980 (0.956–1.000)	0.940	0.950	0.932	0.927	0.953
T1	Test	0.718 (0.490–0.946)	0.714	0.700	0.727	0.700	0.727
T2	Test	0.745 (0.514–0.977)	0.762	0.700	0.818	0.778	0.750
T1 + T2	Test	0.827 (0.613–1.000)	0.857	0.900	0.818	0.818	0.900
T1 + Clinical	Test	0.745 (0.522–0.969)	0.762	0.800	0.727	0.727	0.800
T2 + Clinical	Test	0.800 (0.593–1.000)	0.810	0.800	0.818	0.800	0.818
Fusion	Test	0.891 (0.745–1.000)	0.857	0.800	0.909	0.889	0.833

CI: confidence interval; AUC: area under curve; ACC: accuracy; SEN: sensitivity; SPE: specificity; PPV: positive predictive value; NPV: negative predictive value.

## Data Availability

The raw data supporting the conclusions of this article will be made available by the authors on request.

## Abbreviations

CLNM: central lymph node metastasis; CNN, convolutional neural network; CT, computed tomography; DL, deep learning; ETE, extrathyroidal extension; FNAB, fine needle aspiration biopsy; ML, machine learning; MLP, multilayer perceptron; MRI, magnetic resonance imaging; PET, positron emission tomography; PTC, papillary thyroid cancer; T1WI, T1-weighted imaging; T2WI, T2-wighted imaging; TC, thyroid cancer; US, ultrasound.
